# Investigating the effects of the main agronomic interventions on carabids and spiders in European arable fields: A systematic review protocol

**DOI:** 10.1186/s13750-025-00359-4

**Published:** 2025-04-19

**Authors:** Coralie Triquet, Yvonne Fabian, Philippe Jeanneret

**Affiliations:** Agroecology and Environment, Reckenholzstrasse 191, Agroscope, Zurich, CH-8046 Switzerland

**Keywords:** Farming practice, Cropping system, Agroecology, Biodiversity, Ground-dwelling arthropods

## Abstract

**Background:**

Designing agroecological cropping systems enhancing functional biodiversity and natural pest regulations requires understanding the ecological processes involved, specifically regarding the response of generalist predators. A more precise knowledge of the changes in ground-dwelling communities implied by individual agronomic interventions is needed to make enlightened and consistent choices in the design of such innovative cropping systems. A recent systematic map showed that fertilization, tillage, pesticides use, grazing and mowing are the most studied agronomic interventions regarding their effects on arthropods. The direct and indirect effects of disturbances induced by agronomic interventions on ground-dwelling arthropods in arable fields have been widely investigated, especially for carabids and spiders. However, there is not always a clear pattern outstanding, probably due to antagonistic responses of species with different functional traits. Here, we propose a quantified synthesis on this topic. We will show the impact of the main agronomic interventions in arable fields on the two most studied ground-dwelling predator groups, carabids and spiders, and compare their response (abundance, species richness, taxonomic and functional diversity) in different contexts (crop types and production methods). We will investigate contrasting responses at different taxonomic levels depending on functional traits.

**Methods:**

The evidence will be identified from the recent systematic map on the impacts of agricultural management practices on biodiversity indicator species groups published in 2024. We will select all studies reporting the effect of the most studied agronomic interventions (fertilization, tillage, pesticide application, mowing and grazing) in arable fields (arable crops and temporary grasslands) on carabids and spiders in the map database. A search update will be performed using the search strings used for the systematic map for carabids and spiders, and extracted references will be sorted at title, abstract and full text levels according to the topic of the present work. All selected studies will be critically appraised and a low, medium, or high risk of bias will be assigned to each study. The synthesis of the data extracted from the studies will be first narrative (using qualitative data), and then quantitative for those with adequate data for a meta-analysis.

**Supplementary Information:**

The online version contains supplementary material available at 10.1186/s13750-025-00359-4.

## Background

The intensification of agricultural practices in Europe over the last decades has radically transformed agroecosystems. Specifically, the intensification of land use and pesticides use [1], along with the homogenization of the landscape [2], are the main drivers of arthropod biodiversity loss [3, 4]. This decline raises significant concerns about the loss of essential ecosystem services, such as pollination, pest regulation and the decomposition of organic matter [5]. There is indeed no more doubt about the primary role of arthropods and their associated ecosystem services provision for agricultural production [6, 7].

To turn the tide, agriculture must change rapidly towards agroecology, an approach using ecological concepts and principles for the design and management of more sustainable food systems [8–10]. For this purpose, there is a need to design and experiment innovative agroecological cropping systems that rely on ecological processes and ecosystem services. The main approaches are: decreasing detrimental disturbances linked to agronomic interventions such as pesticide use, inappropriate fertilization and soil operations, and mowing and grazing, increasing crop diversity by the introduction of legumes and crop associations (spatially and temporally through crop rotation) within production fields, and implement more semi-natural infrastructures like hedges and flowering areas [11, 12]. To help farmers adopt this approaches, agri-environmental schemes have been developed in the EU policies, with financial incentives conditioned to the implementation of some agroecological practices, however with limited results on biodiversity loss mitigation to date [13].

To design agroecological cropping systems that enhance functional biodiversity and the delivery of associated ecosystem services (e.g., pest regulation, pollination), it is necessary to understand the ecological processes involved and the spatio-temporal dynamics of arthropod communities in particular. Indeed, a more precise knowledge of the changes in communities implied by individual agronomic interventions is needed to limit negative impacts on beneficial populations and to favor natural pest regulation for instance. This is of prime importance to make enlightened and consistent choices of practices in the design of agroecological cropping systems.

In this context, ground-dwelling arthropods have been widely studied as generalist predators, especially carabids and spiders. These arthropods are indeed usually very abundant in cultivated landscapes and numerous species are adapted to cropped and open habitats. Moreover, they are known to be important ecosystem services providers, by contributing to pest and weed regulation in arable crops [14, 15]. Studies have shown that their taxonomic and functional diversity in cultivated landscapes play a crucial role in the enhancement of natural pest control and contribute to weed regulation in crops [16–18].

A recent systematic map about the effects of farming practices on biodiversity [19] showed that numerous studies have investigated the direct and indirect effects of disturbances induced by farming practices on soil fauna and ground-dwelling arthropods, especially carabids and spiders. Specifically, fertilization, tillage, pesticides use, grazing and mowing are the most studied agronomic interventions regarding their effects on carabids and spiders. However, there is not always a clear pattern, probably due to antagonistic responses of species with different functional traits [20–24]. Quantitative syntheses of these field evidences are now possible and make it possible to draw an overview of the response of carabids and spiders to agronomic interventions [25, 26]. Recent reviews addressed the effect of farming practices on biodiversity in general [27, 28], and the effect of farming intensity and production methods [29], as well as tillage regimes [30] on carabids at the global scale. Here, we propose to answer the need for recent quantified synthesis on this topic, with a systematic review and meta-analysis about the effects of the main agronomic interventions on both carabids and spiders. We will specifically explore the effects of pesticides use, fertilization, mowing, and tillage, on carabids and spiders at different taxonomic and functional levels. By doing so, we intent to (i) quantitatively synthesize the effect of those practices on abundance and diversity of carabids and spiders, indicating if there is a general pattern standing out from the existing evidence, and (ii) explore the different responses of sub-groups (e.g., species, families, functional groups) that could explain unclear results or contradictory outcomes between studies.

The primary question of this systematic review reflects the conclusions of the systematic map published in 2024 (Triquet et al. 2024). Specifically, there is a clear need for in-depth analyses and synthesis of the relationships between farming practices and carabid and spider communities. The formulation of the question and its components was guided by a thorough examination of the matrix combining biodiversity indicators and farming practices by the researchers involved in the systematic map. From this, researchers identified the combinations with reliable information in terms of published results to perform a quantitative analysis.

## Objective of the review

The objective of this review is to investigate the impact of the main agronomic interventions in arable fields on the two most studied ground-dwelling predator groups, carabids and spiders. We want to quantify the impact of these farming practices on the abundance and diversity of these two generalist predators, in different contexts (crop type, production method), to provide indications for the design of innovative agroecological cropping systems. We will investigate in the possibly contrasted responses to farming practices at different taxonomic levels and their relationship to functional traits when reported (e.g., diet, hunting strategy, body size).

### Primary question

The primary question is: What are the effects of the main agronomic interventions in arable fields on carabids and spiders communities?

### Components of the primary question

The key elements of the primary questions based on the PICOc framework are:

- Population: carabids and spiders.
- Intervention: the main agronomic interventions, i.e., fertilization, tillage, pesticide application, mowing and grazing.
- Control: the comparison before-after interventions, or, between intervention and control plots or fields.
- Outcome: measure of change of carabids and spiders, i.e., abundance, species richness, (functional) diversity, (functional) evenness.
- Type of study: all field studies, with a factorial experiment design or an on-farm design, where effects of interventions are assessed directly in arable crops and rotational grasslands.

## Methods

The systematic review will follow the Collaboration for Environmental Evidence Guidelines and Standards for Evidence Synthesis in Environmental Management [31], and will conform to ROSES reporting standards [32] as well as this protocol (see Additional file 1).

### Search for articles

The evidence will be identified from the recent systematic map on the impacts of agricultural management practices on biodiversity indicator species groups, published in 2024 [19]. All articles, including grey literature, in the map database reporting the effect of the agronomic interventions of interest (fertilization, tillage, pesticide application, mowing and grazing) in arable fields (arable crops and temporary grasslands) on carabids and spiders will be selected. This selected sub-set of articles from the systematic map will have to fulfill the eligibility criteria presented hereafter (selection by filters within the database of the systematic map, Additional file 5 [19]).

To identify the evidence produced since then (April 2022), a search update will be performed using the same search string, applied to spiders and carabids only (see Additional file 2, and methods section from the systematic map [19]), as follow (example given for spiders in Web of Science):

TI/AB/AK=(arane* OR arachnid* OR spider$).

AND

TI/AB/AK=(((arane* OR arachnid* OR spider$) NEAR/3 (richness OR composition$ OR abundan* OR diversity OR evenness OR number$ OR assemblage$ OR communit* OR population$)) OR (species NEAR/3 (richness OR composition$ OR abundan* OR diversity OR evenness OR number$ OR assemblage$ OR communit* OR population$)) OR shannon OR simpson).

AND

TI/AB/AK=(“soil preparation” OR till* OR plough* OR fertili* OR amendment* OR compost* OR biochar* OR manur* OR sow* OR planting OR irrigat* OR watering OR “crop protection” OR “pest control” OR “pest management” OR “weed control” OR pesticide$ OR insecticide$ OR herbicide$ OR rodenticide$ OR bactericide$ OR harvest* OR reaping OR “residue management” OR “crop residue” OR mow* OR cutting OR hay OR silage OR grazing OR pasture$ OR husbandry OR livestock$ OR cattle$ OR “cover crop” OR “catch crop” OR “intermediate crop” OR “high nature value” OR hnv$ OR “agri-environment schemes” OR aes OR “semi-natural” OR snh$ OR “ecological compensation” OR eca$ OR “biodiversity promotion” OR bpa$ OR “ecological focus” OR efa$ OR land$use OR organic OR conventional OR agro$ecology OR agro$forestry OR “crop rotation”).

AND

TI/AB/AK=(farm* OR agri* OR crop* OR grassland* OR arable OR cultivated).

AND

ALL=(Albania OR Andorra OR Austria OR Belarus OR Belgium OR Bosnia OR Herzegovina OR Bulgaria OR Croatia OR Cyprus OR Czech* OR Denmark OR Estonia OR Finland OR France OR Germany OR Greece OR Hungary OR Ireland OR Italy OR Kosovo OR Latvia OR Liechtenstein OR Lithuania OR Luxembourg OR Moldova OR Monaco OR Montenegro OR Netherlands OR Macedonia OR Norway OR Poland OR Portugal OR Romania OR “San Marino” OR Serbia OR Slovakia OR Slovenia OR Spain OR Sweden OR Switzerland OR Ukraine OR “United Kingdom” OR “UK” OR England OR Britain OR Scotland OR Wales OR Europe*).

The comprehensiveness of the search string has already been estimated for the systematic map using a test-list of 90 articles, with a hit-score of 87. As for the systematic map, we will search for relevant evidence on the Web of Science Core Collection and CABI platforms, using the institutional access of Agroscope. This will be completed with a search on Google scholar. Only articles written in English will be extracted.

### Article screening and study eligibility criteria

#### Screening process

The screening process of the new references will be performed successively at the title, abstract and full text levels. At the title and abstract levels, records will be classified as relevant, uncertain or irrelevant, based on the eligibility criteria presented hereafter. Irrelevant records will be excluded from the review. Relevant and uncertain records will be passed to the next level of screening and reevaluated. At the full text level, articles are either kept (relevant) or excluded (irrelevant) from the review, and reasons of exclusion for rejected articles will be provided as additional information.

The screening of the newly published articles will be undertaken by one reviewer (CT, except for her own publications if they appear in the extracted references which will be screened by YF or PJ), following the same strategy as for the systematic map for consistency. The repeatability of this process will be tested by the review team by double checking 100 studies at the title level, 100 studies at the abstract level, and 50 articles at the full text level, and by computing kappa tests (see systematic map [19]). For the double checking, YF and PJ will conduct the screening of these 100 titles, 100 abstracts and 50 full texts, accordingly to the eligibility grid (presented hereafter and in Additional file 3) and the results will be compared with CT’s results. If differences occur, decision rules will be chosen together for consistency. No reviewer will assess his/her own publications.

#### Eligibility criteria

To be included in the systematic review, articles must fulfill the following conditions:

- Eligible populations: articles must include carabids and/or spiders.
- Eligible interventions: articles must include at least one of the agronomic interventions, i.e., fertilization, tillage, pesticide application (herbicides, insecticides, molluscicides, and fungicides), mowing or grazing.
- Eligible comparators: articles must compare interventions and controls, in a Control-Impact (CI) or Before-After Control-Impact (BACI) design.
- Eligible outcomes: articles must report measures of abundance (or activity-density), species richness, diversity or evenness.
- Eligible types of study design: articles must be field studies, with a factorial experiment design, on-farm or paired design.
- Eligible crop type: articles must report research conducted in arable fields, specifically arable crops, fodder crops and temporary (rotational) grasslands.
- Eligible location: articles must report studies conducted on the European mainland.
- Eligible language: articles must be written in English.

We developed a detailed version of this list of eligibility criteria to guide and standardize the literature screening, available in Additional file 3. Articles that will be excluded based on that list at full text screening stage will be provided as additional information.

### Study validity assessment

Each study fulfilling the eligibility criteria presented above will be assessed for both internal validity (risk of selection, performance, detection, and attribution bias) and external validity (the degree to which the studies are in adequation with the review topic and appropriate to answer the review primary question). The internal and external validity will be evaluated by attributing a low, medium, or high risk of bias. To do so, we will use a questionnaire of 12 questions adapted from Meissle et al., 2022 (data file 3 [33]),.

Internal validity:

- Is the spatial dispersion of replicated fields or plots homogeneous, i.e. not grouped by treatment in space?
- Is the number of replicates sufficient to ensure reliable statistical analyses? (the different cut-off values are given in Additional file 4)
- Is the history of field management (before the start of the experiment) similar between treatment and control?
- Is the field management (besides the tested agronomic interventions) similar between treatment and control, or, are there confounding effects due to differences in the cropping system?
- Is the crop the same for treatment and control?
- Is the monitoring method (for carabids and spiders) similar between treatment and control?
- Is the sampling size (and amount of missing data) equal for treatment and control?

External validity:

- Were insecticides used during the experiment (besides for articles testing the effect of insecticides)?
- Is the monitoring method used adequate and commonly used for the taxon and life stages studied?
- Is the sampling repeated over time? (Number of samplings per growing season)
- Is the taxon collected in sufficient number? (Number of individuals)
- Are there other major risks of bias, or, does the data seem reliable?

The questions are adapted for the two types of design we identified based on the units of replication: A - factorial experiment design (units are plots within a field), and B - on-farm design (units are individual fields, paired or not in the landscape). For each question, responses (including cut-off values and cases of unreported information) are attributed to low, medium or high risk of bias, and the global risk of bias attributed for one study will be medium if at least one medium risk was identified, and high if at least one high risk has been identified. In some cases that are detailed in Additional file 4, the absence of information or a high risk score leads to the exclusion of the article from the study (e.g., crop species or number of replicates not given). We developed a grid to guide and standardize the study validity assessment of the selected articles, available in Additional file 4. The results of the study validity assessment will be provided as additional information.

The study validity assessment of every included study will be undertaken by one reviewer (CT, except her own publications), the repeatability of this process will be ensured by double checking of each study by either YF or PJ. If differences occur, decision rules will be chosen together for consistency. No reviewer will assess his/her own publications.

The results of the critical appraisal will be used to conduct sensitivity analysis. In both the narrative and the quantitative synthesis, the results of studies with low risk of bias will be first synthesized, and then the results of studies with medium and high risk of bias will be considered, and conclusions will be compared.

### Data coding and extraction strategy

The metadata extracted from the selected articles will include bibliographic information, study design, crop type, carabids and/or spiders measured outcomes (e.g., abundance, richness, diversity and evenness), monitoring methods used, and the agronomic intervention studied. These metadata have already been extracted from the articles included in the original systematic map [19], and will be extracted using the same method for the new references. Additionally, we will extract the following information that could be useful for the analyses of the data or validity assessment: production method, number of replicates per treatments, number of samples per replicates, number of individuals per replicate.

For each study, one or several effects will be recorded (represented by one line in the database). One effect is the combination of one outcome and one intervention, and will be translated into an effect size by comparison between a treatment and a control. For each recorded effect, quantitative data will be extracted from text, tables and figures using the package metaDigitise [34] in the R environment [35]. The quantitative data extracted will be sample size, mean and a measure of variation of the mean (standard deviation, standard error, coefficient of variation) for both the control and the treatment. Qualitative results (significance and direction of the effect) will be extracted, even for articles that do not provide the needed quantitative data.

The data extraction strategy will depend on agronomic interventions considered, resulting in 1 database for metadata of articles, and 4 databases of quantitative and qualitative data (1 for tillage, 1 for fertilization, 1 for pesticides use, and 1 for grazing and mowing together), that will be provided in additional files of the systematic review. The data coding spreadsheets and rules for extraction of data are available in Additional File 5. For articles with missing quantitative data (including in the supplementary material and published datasets), authors will be contacted by e-mail in order to obtain them. Authors of studies with unclear or missing information concerning data will be contacted too.

Data coding and extraction from the selected studies will mainly be undertaken by one reviewer (CT), the repeatability of this process will be tested by the review team by double checking 5% of the studies. If differences occur, decisions rules will be chosen together for consistency.

### Potential effect modifiers/reasons for heterogeneity

Based on the expertise of the review team, the following potential effect modifiers will be considered:

- Crop type (cereals, maize, row crops, oleaginous, cover crops and rotational grasslands that are included in the eligibility list in Additional file 3).
- Production method (conventional, organic, conservation agriculture).
- Temporal scale of intervention (time since application of the treatment: short term, one year, 2–5 years, or long term).
- Spatial scale of intervention (field, farm, or landscape scale).
- Monitoring method (e.g., pitfall traps, emergence tents, sweep net).
- For fertilization: type (organic, mineral) and dose.
- For tillage: intensity (minimal, non-inversion, conventional ploughing), depth and frequency.
- For pesticides use: type (herbicide, insecticide, fungicides, all), dose or Indicator of Frequency of treatment (IFT).
- For mowing and grazing: frequency, intensity (number of animals / hectare).

### Data synthesis and presentation

In a first step, the studies selected for the review and their results will be described in a narrative synthesis, i.e., a qualitative description of the studies and their results using vote counting (significance and direction of the effects recorded as positive, neutral or negative). Then, only studies with the adequate quantitative data (sample size, mean and measure of variation of the mean for both the control and the treatment, or technical data to estimate them) will be included in the quantitative synthesis. The statistical analyses will be conducted in the R environment [35] using the package metafor [36]. We will calculate effect sizes for each study and apply a random-effects model to synthesize the effect sizes [35]. The results will be synthesized by agronomic intervention, and highlighted by the different modifiers (see above), in particular crop type and production method of the cropping system. To do so we will conduct one meta-analysis for each intervention, for both carabids and spiders. If the data allow it, we will also conduct these meta-analyses on sub-groups (e.g., genus, species, functional group), in order to investigate the potential contrasted responses at different taxonomic and functional levels. The resulting meta-analyses and the rules for the control and intervention(s) for each of these will be detailed further after the exploration of the data, but are planned as followed for now:

- For tillage, 1 meta-analysis will compare “conventional inversion tillage” (deep ploughing) as control with “non-inversion tillage” (often called minimum tillage), 1 meta-analysis will compare “conventional inversion tillage” as control with “no tillage” and/or “conservation agriculture” (grouped if relevant, or separated if the no-tillage studies did not change any other practice).
- For fertilization, 1 meta-analysis will compare “no fertilization” as control with a fertilized treatment (nitrogen fertilization), taking the highest dose when several doses were tested.
- For pesticides use, 1 meta-analysis will compare “no pesticide” as control with a treatment with pesticides, taking the highest dose when several doses were tested, and using the pesticide type as a moderator.
- For mowing or grazing, 1 meta-analysis will compare “no grazing/mowing” as control with a mowed/grazed treatment, taking the highest intensity/frequency when several were tested. If there is enough studies, we will separate mowing and grazing in two distinct meta-analyses.

In both the narrative and the quantitative synthesis, the results of studies with low risk of bias will be first synthesized, and then the results of studies with medium and high risk of bias will be considered. However, in some cases, a high risk of bias in one question leads to the exclusion of the paper from the review (see study validity assessment in red Additional file 4), thus the concerned studies will not be included in the analyses at all. Potential publication bias will be checked by visual inspection of funnel plots [35] and analysis of predicted values (predictive model including an estimated number of missing studies).

## Electronic supplementary material

Below is the link to the electronic supplementary material.

Supplementary Material 1: ROSES report for systematic review protocol

Supplementary Material 2: Search strings

Supplementary Material 3: Eligibility criteria

Supplementary Material 4: Study validity assessment

Supplementary Material 5: Data coding strategy

## Data Availability

No datasets were generated or analysed during the current study.
