# Supplementary material for: Investigating the effects of the main agronomic interventions on carabids and spiders in European arable fields: A systematic review protocol
Source: Environ Evid. 2025 Apr 19;14:6. doi: 10.1186/s13750-025-00359-4 (PMC12008925; doi:10.1186/s13750-025-00359-4)
Supplement: Supplementary file 1 — Supplementary Material 1: ROSES report for systematic review protocol [file 13750_2025_359_MOESM1_ESM.pdf]

Item number	Section / sub-section	Topic	Description	Further explanation	Checklist/Meta-data	Author response
1	Title	Title	[illegible] indicate if it is an update/amendment: e.g. "A systematic review update protocol...".	The title should normally be the same or very similar to the review question.	Meta-data	What are the effects of the main agronomic interventions on carabids and spiders in European arable fields? A systematic review protocol
2	Type of review	Type of review	Select one of the following types of review: systematic review, systematic	See CEE Guidance on amendments and	Meta-data	systematic review from a systematic map
3	Authors contacts	Authors contacts	The full names, institutional addresses, and email addresses for all authors		Checklist	Yes
4	Abstract	Structured summary	Abstract must not exceed 350 words and must include two sections 1)		Checklist	Yes
5	Background	Background	Describe the rationale for the review in the context of what is already	A theory of change and/or conceptual	Checklist	Yes
6	Stakeholder engagement	Stakeholder engagement	The planned/actual role of stakeholders throughout the review process		Checklist	Yes
7	Objective of the review	Objective	Describe the primary question and secondary questions (when applicable).	The primary question is the main	Checklist	Yes
8		Definitions of the question components	Break down and summarise question key elements e.g. population, intervention(s)/exposure(s), comparator(s), and outcome(s).	For other question types see [3,4]	Meta-data	•Population: carabids and spiders.<br>•Intervention: the main agronomic interventions, i.e., fertilization, tillage, pesticide application, mowing and grazing.<br>•Control: the comparison before-after interventions between intervention and control plots or fields.<br>•Outcome: measure of change of carabids and spiders, i.e., abundance, species richness, (functional) diversity, (functional) evenness .<br>•Type of study: all field studies, with a factorial experiment design or an on-farm design, where effects of interventions are assessed directly in arable crops and rotational grasslands.
	Methods					
9	Searches	Search strategy	Detail the planned search strategy to be used, including: database names	Details regarding search strategy testing	Checklist	Yes
10		Search string	Provide Boolean-style full search string and state the platform for which the string is formatted (e.g. Web of Science format)		Meta-data	TI/AB/AK=(arane* OR arachnid* OR spider$)<br>AND<br>TI/AB/AK=(((arane* OR arachnid* OR spider$) NEAR/3 (richness OR composition$ OR abundan* OR diversity OR evenness OR number$ OR assemblage$ OR communit* OR population$)) OR (species NEAR/3 (richness OR composition$ OR abundan* OR diversity OR evenness OR number$ OR assemblage$ OR communit* OR population$)) OR shannon OR simpson)<br>AND<br>TI/AB/AK=("soil preparation" OR till* OR plough* OR fertili* OR amendment* OR compost* OR biochar* OR manur* OR sow* OR planting OR irrigat* OR watering OR "crop protection" OR "pest control" OR "pest management" OR "weed control" OR pesticide$ OR insecticide$ OR herbicide$ OR rodenticide$ OR bactericide$ OR harvest* OR reaping OR "residue management" OR "crop residue" OR mow* OR cutting OR hay OR silage OR grazing OR pasture$ OR husbandry OR livestock$ OR cattle$ OR "cover crop" OR "catch crop" OR "intermediate crop" OR "high nature value" OR hnv$ OR "agri-environment schemes" OR aes OR "semi-natural" OR snh$ OR "ecological compensation" OR eca$ OR "biodiversity promotion" OR bpa$ OR "ecological focus" OR efa$ OR land$use OR organic OR conventional OR agro$ecology OR agro$forestry OR "crop rotation")<br>AND<br>TI/AB/AK=(farm* OR agri* OR crop* OR grassland* OR arable OR cultivated)<br>AND<br>ALL=(Albania OR Andorra OR Austria OR Belarus OR Belgium OR Bosnia OR Herzegovina OR Bulgaria OR Croatia OR Cyprus OR Czech* OR Denmark OR Estonia OR Finland OR France OR Germany OR Greece OR Hungary OR Ireland OR Italy OR Kosovo OR Latvia OR Liechtenstein OR Lithuania OR Luxembourg OR Moldova OR Monaco OR Montenegro OR Netherlands OR Macedonia OR Norway OR Poland OR Portugal OR Romania OR “San Marino” OR Serbia OR Slovakia OR Slovenia OR Spain OR Sweden OR Switzerland OR Ukraine OR “United Kingdom” OR “UK” OR England OR Britain OR Scotland OR Wales OR Europe*)
11		Languages – bibliographic	List languages to be used in bibliographic database searches.		Meta-data	english
12		Languages – grey literature	List languages to be used in organizational websites searches and web-		Meta-data	english, french
13		Bibliographic databases	Provide the number of bibliographic databases to be searched.		Meta-data	2
14		Web – based search engines	Provide the number of web – based search engines to be searched.		Meta-data	1
15		Organisational websites	Provide the number of organisational websites to be searched.		Meta-data	3
16		Estimating the comprehensiveness	Describe the process by which the comprehensiveness of the search		Checklist	Yes
17		Search update	Describe any plans to update the searches during the conduct of the	Optional. A search update is good	Checklist	Yes
18	Article screening and study	Screening strategy	Describe the methodology for screening articles/studies for		Checklist	Yes
19		Consistency checking	Describe clearly the process for checking consistency of decisions including		Checklist	Yes
20		Inclusion criteria	Describe the inclusion criteria used to assess relevance of identified		Checklist	Yes
21		Reasons for exclusion	State that you will provide a list of articles excluded at full text with		Checklist	Yes
22	Critical appraisal	Critical appraisal	Describe here the method you propose for critical appraisal of study		Checklist	Yes
23		Critical appraisal strategy	Describe how the information from critical appraisal will be used in		Checklist	Yes
24		Consistency checking	Describe how repeatability of critical appraisal of study validity will be		Checklist	Yes
25	Data extraction	Meta-data extraction and coding	Describe the method for meta-data extraction and coding for studies		Checklist	Yes
26		Data extraction strategy	Describe the method for extraction of qualitative and/or quantitative		Checklist	Yes
27		Approaches to missing data	Describe any processes for obtaining and confirming missing or unclear		Checklist	Yes
28		Consistency checking	Describe how repeatability of the meta-data/data extraction process will		Checklist	Yes
29	Potential effect modifiers/reasons	Potential effect modifiers/reasons	Provide a list of and justification for the effect modifiers /reasons for	The list should not be exhaustive but a	Checklist	Yes
30	Data synthesis and presentation	Data synthesis and presentation	State the type of synthesis conducted as part of the systematic review		Meta-data	Narrative synthesis, qualitative map, meta-analysis
31		Narrative synthesis strategy	Describe methods to be used for narratively synthesising the evidence	Vote-counting (tallying of studies based	Checklist	Yes
32		Quantitative synthesis strategy	If data are appropriate for quantitative synthesis, describe planned	Compulsory if appropriate for data	Checklist	Yes
33		Qualitative synthesis strategy	Describe methods to be used for synthesising qualitative data and justify	Compulsory if appropriate for data	Checklist	Yes
34		Other synthesis strategies	Describe any other approaches to be used for synthesising data or	Compulsory if appropriate for data	Checklist	n/a
35		Assessment of risk of publication	Describe planned methods for examining the possible influence of	For quantitative syntheses this may be	Checklist	yes
36		Knowledge gap identification	Describe the methods to be used to identify and/or prioritise key	Optional	Checklist	n/a
37		Demonstrating procedural	Describe the role of systematic reviewers (who have also authored articles	Reviewers who have authored articles	Checklist	Yes
38	Declarations	Competing interests	Describe of any financial or non-financial competing interests that the		Checklist	Yes

References

[1] Bayliss, H.R., Haddaway, N.R., Eales, J., Frampton, G.K. and James, K.L., 2016. Updating and amending systematic reviews and systematic maps in environmental management. Environmental Evidence, 5(1), p.20.

[2] Haddaway, N.R., Kohl, C., da Silva, N.R., Schiemann, J., Spök, A., Stewart, R., Sweet, J.B. and Wilhelm, R., 2017. A framework for stakeholder engagement during systematic reviews and maps in environmental management. *Environmental Evidence* , *6* (1), p.11.

[3] Collaboration for Environmental Evidence. 2018. Guidelines and Standards for Evidence synthesis in Environmental Management. Version 5.0. www.environmentalevidence.org/information-for-authors.

[4] Leeds Institute of Health Sciences. https://medhealth.leeds.ac.uk/info/639/information_specialists/1500/search_concept_tools. Accessed 12/11/2017.
